# A lesson in business: cost-effectiveness analysis of a novel financial incentive intervention for increasing physical activity in the workplace

**DOI:** 10.1186/1471-2458-13-953

**Published:** 2013-10-10

**Authors:** Mary Anne T Dallat, Ruth F Hunter, Mark A Tully, Karen J Cairns, Frank Kee

**Affiliations:** 1 Centre for Public Health, Queen’s University Belfast, Institute of Clinical Sciences B, Royal Victoria Hospital, Grosvenor Road, Belfast, Northern Ireland, UK; 2 Department of Epidemiology and Biostatistics, Memorial Sloan-Kettering Cancer, Research Center, New York, NY 10065, USA; 3 UKCRC Centre of Excellence for Public Health, Queens University Belfast, Institute of Clinical Sciences B, Royal Victoria Hospital, Grosvenor Road, Belfast, Northern Ireland, UK; 4 Centre for Statistical Science and Operational Research (CenSSOR), Queen's University Belfast, Belfast, Northern Ireland, UK

**Keywords:** Physical activity, Cost-effectiveness analysis, Financial incentives, Workplace intervention

## Abstract

**Background:**

Recently both the UK and US governments have advocated the use of financial incentives to encourage healthier lifestyle choices but evidence for the cost-effectiveness of such interventions is lacking. Our aim was to perform a cost-effectiveness analysis (CEA) of a quasi-experimental trial, exploring the use of financial incentives to increase employee physical activity levels, from a healthcare and employer’s perspective.

**Methods:**

Employees used a 'loyalty card’ to objectively monitor their physical activity at work over 12 weeks. The *Incentive Group* (n=199) collected points and received rewards for minutes of physical activity completed. The *No Incentive Group* (n=207) self-monitored their physical activity only. Quality of life (QOL) and absenteeism were assessed at baseline and 6 months follow-up. QOL scores were also converted into productivity estimates using a validated algorithm. The additional costs of the *Incentive Group* were divided by the additional quality adjusted life years (QALYs) or productivity gained to calculate incremental cost effectiveness ratios (ICERs). Cost-effectiveness acceptability curves (CEACs) and population expected value of perfect information (EVPI) was used to characterize and value the uncertainty in our estimates.

**Results:**

The *Incentive Group* performed more physical activity over 12 weeks and by 6 months had achieved greater gains in QOL and productivity, although these mean differences were not statistically significant. The ICERs were £2,900/QALY and £2,700 per percentage increase in overall employee productivity. Whilst the confidence intervals surrounding these ICERs were wide, CEACs showed a high chance of the intervention being cost-effective at low willingness-to-pay (WTP) thresholds.

**Conclusions:**

The Physical Activity Loyalty card (PAL) scheme is potentially cost-effective from both a healthcare and employer’s perspective but further research is warranted to reduce uncertainty in our results. It is based on a sustainable “business model” which should become more cost-effective as it is delivered to more participants and can be adapted to suit other health behaviors and settings. This comes at a time when both UK and US governments are encouraging business involvement in tackling public health challenges.

## Background

On account of the associated morbidity from related chronic conditions, the current physical inactivity pandemic is placing a huge financial burden on healthcare systems [1]. Estimated annual direct health-care costs related to physical inactivity for the UK are £0.9 billion [2] and including indirect costs, $251.11 billion in the USA [3]. Public health interventions that improve levels of physical activity may thus improve health and wellbeing and combat rising healthcare costs. Due to pressure on healthcare budgets, these interventions must be cost-effective, with the potential for sustained behavior change through the implementation of large scale, long-term effective interventions.

Recently both the UK and US governments have advocated the use of financial incentives to encourage healthier lifestyle choices [4,5]. To date, financial incentives have been found to be most effective within the areas of substance abuse and smoking cessation [6-8]. Their use in improving physical activity levels has been less extensively investigated but a small number of studies exist, showing significant improvements in physical activity and/or participation in physical activity programs, using 'modest’ financial incentives [9-12]. However, irrespective of their behavioral focus there is a dearth of evidence on the cost-effectiveness of financial incentives for behavior change [13]. Previous schemes have also tended to be very costly due to the ongoing cost of the monetary incentive given [7]. For example, one smoking cessation program offered participants a total of $750 if they abstained from smoking for 12 months [14]. In publicly funded healthcare systems such as the National Health Service in the UK, these schemes may be neither sustainable nor, in some people’s views, ethical because of their opportunity cost i.e. the loss of potential gain from other interventions not able to be funded. Therefore if financial incentive schemes are to be implemented in the long-term, they need to be based on a sustainable model.

Much can be learned from the retail sector, which has long been successful at influencing sustained customer behaviors through various incentive schemes. For example, the loyalty or reward card market in the UK is one of the most prominent in the world [15]. Loyalty card schemes are used to reward, and therefore encourage, repeated buying behavior by either entitling the customer to a discount on their goods or allocating them “points” from money spent which can be used to buy future goods or services. They are designed to attract and maintain customers through incentives and in turn influence long term, repeated behavior. It is contended that similar methods could be employed in public health to influence 'healthy’ sustainable behavior change [16]. Further, by fostering links with local businesses, encouraged by the UK Public Health Responsibility Deal, [4] a sustainable model could be established by local businesses sponsoring financially incentivized public health programs. Indeed the UK Public Health Responsibility Deal was introduced in 2011 by the current UK government for the specific purpose of increasing business involvement in tackling public health challenges [4].

The Physical Activity Loyalty (PAL) card scheme was a quasi-experimental study where employees from a workplace setting were each given a loyalty card to monitor their physical activity levels, by swiping their card at receivers placed along designated walking routes, within the grounds of their workplace. Participants were randomly allocated to either an *Incentive* or *No Incentive group* and both groups were able to obtain real-time feedback on their completed physical activity by logging onto the study website. However, for the *Incentive Group*, minutes of physical activity were also converted into points and these points could be redeemed for rewards sponsored by local businesses [16]. The study found positive results for 'modest’ financial incentives on physical activity levels [12]. The aim of this current study is to investigate the cost-effectiveness of the PAL study at increasing physical activity levels. A cost-effectiveness analysis (CEA) from both a healthcare and employer’s perspective will be performed with outcomes measured in quality adjusted life years (QALYs) and gains in productivity, respectively, to highlight the 'returns’ for both providers.

## Methods

The PAL study was a researcher blinded quasi-experimental trial. Participants were recruited from an office-based workplace and followed up over 6 months. Details of the study design, the intervention and its effectiveness on physical activity have been previously published [12]. The study was approved by the School of Medicine, Dentistry and Biomedical Sciences Ethics Committee, Queen’s University Belfast, Northern Ireland.

### Recruitment

Employees working in two large buildings at Northern Ireland’s main government offices (which are set in over 300 acres of parkland) were recruited via email invitation, posters and a web link on relevant intranet sites. Eligibility criteria included those aged 16–65 yrs old, based at their office ≥4 days/week and ≥6 hours/day, and able to complete 15 minutes of moderate paced walking (self-reported). Interested participants were directed to the study website where they could access further information, register to participate and complete a screening questionnaire. Eligible participants then provided informed consent, were given a PAL card and asked to complete a baseline questionnaire [12].

### Allocation

A computer-generated random allocation sequence was prepared by a statistician not involved in the administration of the trial and random assignments were placed in individually numbered, sealed envelopes by the statistician to ensure concealment of allocation. All eligible participants in Building A were randomly allocated (grouped by building to reduce contamination) to group 1: the *Incentive Group* and those in Building B were randomly allocated to group 2: the *No Incentive Group*[12].

### Intervention

When undertaking physical activity during their working day over the 12 week intervention period, participants scanned their PAL card, containing a passive Radio Frequency Identification (RFID) tag, at Near-Field Communication (NFC) sensors positioned along walking routes and at the entrance to a gym and exercise studio, within the grounds of the workplace. The average distance between sensors was 643 meters (min 269 m, max 1017 m). Each time a participant swiped their card, a timestamp was created, recording the date and time of each swipe. The minutes between each timestamp were then aggregated, to give the total minutes of physical activity for each bout completed. Participants could obtain real-time feedback on various aspects of their physical activity, including minutes of physical activity, by logging into their personal account on the study website. A detailed explanation about the web-based technology used has been described elsewhere [17].

Participants from both groups could use their PAL cards to self-monitor their physical activity levels at work. However, for the *Incentive Group*, minutes of physical activity were also converted into points (1 minute = 1 point; capped at 30 points per day) and these points could be redeemed for rewards (retail vouchers) at week 6 and 12. Additional file 1: Table S1 lists the rewards, their points’ value and corresponding monetary value. A marketing consultant was hired to act as an intermediary with the local business sector and was able to negotiate the provision of these vouchers 'in kind’ from local retailers.

### Outcome measures

At baseline, age, gender, self-report height and weight, highest level of education, staff grade and self-report physical activity levels using the Global Physical Activity Questionnaire (GPAQ) [18] were recorded.

For the purposes of this economic evaluation the main outcome measures of interest were objective physical activity (recorded using the PAL cards) which was recorded continuously over the 12 week intervention period, quality of life (QOL) (measured using the weighted health index from EQ-5D) at 6 months follow-up, [19] and self-reported work absenteeism within the past 6 months. Self-reported absenteeism data has been found to strongly correlate with recorded absenteeism data [20].

EQ-5D is a standardized instrument used to measure health status [19]. It comprises two parts- a descriptive system and a visual analogue scale. The descriptive system has five dimensions and each dimension has three levels. By using a formula which attaches weights to each of these levels a single summary health index is produced which can be used in economic evaluations to measure health benefits.

In addition, EQ-5D scores were converted into productivity estimates using a recently developed algorithm [21]. Since QOL can be an indication of someone’s degree of health or illness and different levels of health/illness lead to different levels of productivity then it has been suggested that QOL could be used as a proxy for productivity [22]. Therefore, by utilizing studies demonstrating the relationship between QOL and productivity, researchers have developed an algorithm to translate changes in QOL into quantifiable changes in productivity [21,23]. The algorithm combines two equations which predict an individual’s level of absenteeism and presenteeism, based on their EQ-5D scores, to give an overall productivity estimate between zero and one.

### Statistical analyses

At baseline, groups were compared using Independent Samples T tests and Chi-square tests. Statistical Package for Social Sciences (SPSS) version 17.0 Software for Windows (SPSS Inc, Chicago, USA) was used for data analysis.

We used the previously reported ANCOVA analyses comparing differences between groups in minutes of physical activity at week 6 and 12 and self-reported work absenteeism at 6 months [12]. For QOL and productivity we calculated the differences from baseline to 6 months for each group and used Independent Samples T tests to test if the mean differences between groups were significant.

### Cost-effectiveness analyses

The aim was to present the incremental cost-effectiveness ratio (ICER) of the *Incentive Group* compared to the *No Incentive Group* by identifying the additional costs associated with the *Incentive Group* per additional unit of health outcome (QALY) or percentage gain in productivity. All costs and benefits accrued beyond one year should be discounted to reflect their present value but since our data were collected over 6 months, no discounting was required [24]. All costs were derived in pounds sterling (£).

The itemized costs for the PAL study are listed in Additional file 2: Table S2. The greatest expense common to both groups was that of the research fellow who was responsible for overseeing the PAL scheme. They were involved in the website design, created all content for its inclusion and delivered the swipe cards and vouchers. Of note, the management of participants through the trial was performed automatically by the website thereby reducing implementation costs. The additional costs accrued by the *Incentive Group*, and hence included in the ICER calculations, was due to the cost associated with the marketing consultant hired to negotiate the retail vouchers 'in kind’ and the cost of delivering the rewarded vouchers.

#### Healthcare perspective

EQ-5D scores for every participant at baseline and at 6 months were converted into individual utility scores using the EQ-5D scoring formula [25]. By totaling individual utility gains over 6 months for each group and multiplying by 0.5 years, the total QALYs gained per group was determined. This allowed the difference in QALYs gained between the groups to be calculated. Finally, to calculate the ICER, the additional costs were divided by the additional QALYs gained by the *Incentive Group*.

#### Employer’s perspective

Individual EQ-5D scores at baseline and 6 months were also converted into estimated levels of productivity using a previously validated algorithm [21] and the total productivity gain over 6 months for each group was calculated. To calculate the ICER, from an employer’s perspective, the additional costs were divided by the additional gain in total employee productivity by the *Incentive Group*.

### Sensitivity analysis

We allowed all costs in the study to vary by +/- 5% except that of the research fellow, in which case, we employed the standard salary range that is typical for such a position. All itemized costs for each group were then selected at random from a triangular distribution, approximately 20,000 times, in a Monte Carlo simulation and aggregated. For the individual utility and productivity gains, we used bootstrapping to repeatedly sample individual gains [26]. Cost-effectiveness acceptability curves (CEACs) were used to present the probability that the *Incentive Group* was cost-effective compared to the *No Incentive Group,* from both a healthcare and employer’s perspective, for a range of willingness-to-pay (WTP) thresholds. The WTP threshold represents the maximum amount a decision maker is willing to pay to obtain a unit of health outcome [27]. Although the National Institute for Health and Care Excellence (NICE) in the UK eschews publishing a definite threshold, its past decisions imply a threshold of circa £30,000/QALY ($50,000/QALY) [28].

### Value of information analysis

Value of information analysis can be used to establish the value of additional evidence through further research or equivalently the expected costs of uncertainty for the healthcare sector [29]. We used the Monte Carlo simulation outputs to estimate the individual expected value of perfect information (EVPI) by calculating the difference between the expected value of the decision made with perfect information and the decision made with current evidence. Population EVPI is calculated by multiplying individual EVPI by the total population who could potentially benefit from such a project by the number of years they would be expected to benefit [30]. We chose a conservative assumption where we assumed the project to benefit all current Northern Ireland employees (695,000) [31] for one year and plotted population EVPI for various WTP thresholds. Population EVPI essentially represents the maximum amount of money any healthcare provider would be expected to invest in further research to eliminate uncertainty in the decision about whether the PAL scheme is cost-effective or not. This then allows for prioritization of research as the projects which are expected to achieve the greatest payoff in expected net benefit by obtaining further information can be pursued [32].

## Results

### Baseline characteristics

84% of employees in both groups completed the study at 6 months follow-up. Table 1 shows the baseline characteristics of the participants stratified by group. The mean age of participants was 43.32 ± 9.37 years (mean ±SD), 67% were female and 53% were categorized as having 'low’ physical activity levels at baseline.

**Table 1:** **Baseline characteristics of participants according to group (mean ± 95% CI)**[12]

	***Incentive Group *( *n *=199)**	***No Incentive Group *( *n *=207)**	***p *value**
Age (yrs)	43.30 (SD 9.58)	43.34 (SD 9.20)	0.96
Gender	66% female	68% female	0.78
BMI (kg/m^2^)	27.16 (26.34, 28.02)	26.92 (26.28, 27.54)	0.71
^a^Staff Grade	4.5% (Grade 5+)	3.9% (Grade 5+)	0.07
^a^Education (highest qualification)	40.2% University degree or higher	38.2% University degree or higher	0.97
^a^GPAQ: Physical activity category	20.1% High	20.8% High	0.19
	23.1% Moderate	30.4% Moderate	
	56.8% Low	48.8% Low	
EQ-5D: Weighted Health Index	0.89 (0.87, 0.92)	0.92 (0.90, 0.94)	0.09
Work absenteeism: sick days (in past 6 months)	2.54 (1.23, 3.84)	3.24 (1.60, 4.88)	0.47
Productivity	0.63 (0.62, 0.64)	0.64 (0.63, 0.65)	0.11

Independent Samples T test for normally distributed continuous variables.

^a^Chi-square test for discrete variables.

CI, Confidence Interval.

SD, Standard deviation.

Grade 5+ is the highest staff grade.

### Outcomes

At week 6 the *Incentive Group* performed more minutes of physical activity/week (26.18 mins; 95% CI 20.06, 32.29) than the *No Incentive Group* (24.00 mins; 95% CI 17.45, 30.54) but the difference was not significant (p=0.45). Similarly, at week 12 the *Incentive Group* performed more physical activity/week (17.52 mins; 95% CI 12.49, 22.56) than the *No Incentive Group* (16.63 mins; 95% CI 11.76, 21.51) but again the difference was not significant (p=0.59). From EQ-5D responses, the *Incentive Group* reported greater gains in utility at 6 months (0.03; 95% CI 0.01, 0.05) compared to the *No Incentive Group* (0.02; 95% CI 0.00, 0.04) although these were not significant (p=0.40). After converting EQ-5D scores into productivity estimates, employees in the *Incentive Group* improved their level of productivity over 6 months more (1.13%; 95% CI 0.34%, 1.92%) than the employees within the *No Incentive Group* (0.5%; 95% CI -0.39%, 1.39%) but the difference was not significant (*p*=0.30). Work absenteeism rates were not significantly different between groups at 6 months (*p*=0.22).

### Cost-effectiveness analysis

Mean incremental costs, incremental effects and ICERs, from both a healthcare and employer’s perspective, are displayed in Table 2. The *Incentive Group* cost more than the *No Incentive Group* but it was also more effective resulting in a mean ICER of £2,900/QALY (95% CI -28,000, 32,000) and £2,700/percentage gain in productivity (95% CI -20,000, 30,000).

**Table 2:** Incremental cost-effectiveness ratio calculations (mean +/- 95% CIs)

	**Group 1: *Incentive Group***	**Group 2: *No Incentive Group***	**Difference (Group 1 – Group 2)**	**ICER (£/QALY)**	**ICER (£/% productivity)**
Total costs	£30,800 (30,200, 31,400)	£26,700 (26,200, 27,200)	£4,100 (3,900, 4,300)	£2,900 (-28,000, 32,000)	^a^£2,700 (-20,000, 30,000)
Total QALYs gained	2.4 (0.9, 4.0)	1.2 (-0.3, 2.7)	1.2 (-0.9, 3.4)		
Total productivity gained	1.9 (0.6, 3.1)	0.5 (-0.8, 2.0)	1.3 (-0.5, 3.2)		

^a^Median +/- 95% CIs.

ICER, Incremental cost-effectiveness ratio.

CI, Confidence interval.

QALYs, Quality adjusted life years.

%, percentage.

### Sensitivity analysis

**Figure 1:**
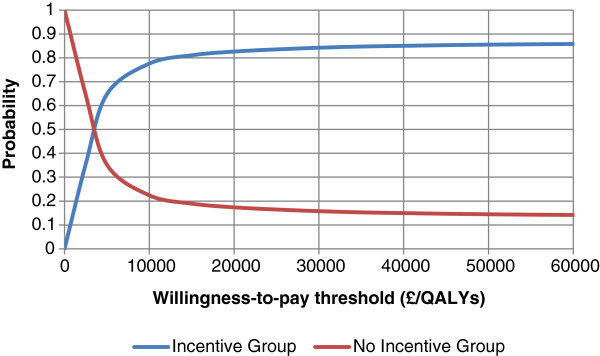
Cost-effectiveness acceptability curves for QALYs gained for the Incentive and No Incentive Group.

**Figure 2:**
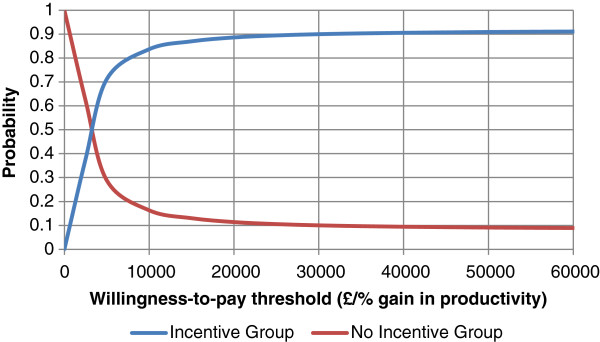
Cost-effectiveness acceptability curves for productivity gained for the Incentive and No Incentive Group.

Figure 1 shows a CEAC showing that at the cost-effectiveness threshold of £30,000/QALY the probability that the *Incentive Group* is cost-effective compared to the *No Incentive Group* is greater than 85%. No cost-effectiveness threshold exists for the business sector but from the CEAC in Figure 2 we see that when an employer is willing to pay at least £4,000 per percentage increase in productivity the probability that the *Incentive Group* is cost-effective compared to the *No Incentive Group* is approximately 50%.

### Value of information analysis

**Figure 3:**
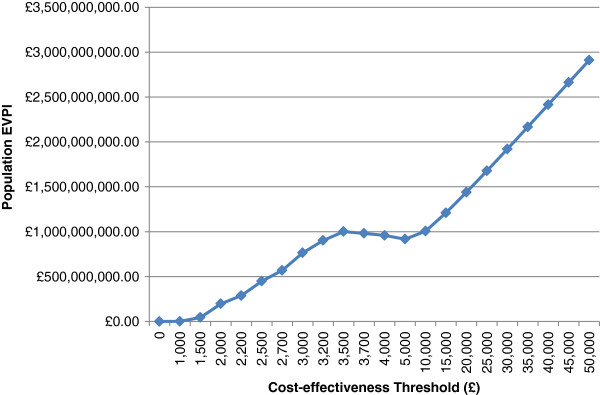
Population expected value of perfect information for the Northern Ireland employee population.

Figure 3 shows the EVPI for the Northern Ireland employee population which is very high (£ billions) even at low WTP thresholds. It continues to increase as the WTP threshold increases indicating that the increase in the monetary consequences of error exceed the reduction in probability of error as the WTP threshold increases. This result suggests that further research is warranted since the cost of further research would almost certainly be less than the EVPI at the £30,000/QALY WTP threshold.

## Discussion

### Healthcare perspective

The PAL scheme is an innovative physical activity loyalty card scheme for behavior change. By applying the techniques of cost-effectiveness analysis we found the intervention could be cost-effective at improving physical activity levels in predominantly inactive, office-based employees from a healthcare perspective. The ICER for the *Incentive Group* compared to the *No Incentive Group* was £2,900/QALY at 6 months which is well below the notional UK cost-effectiveness threshold. Therefore, whilst the additional QALYs gained by the *Incentive Group* was not statistically significant, this difference was enough to offset the additional costs of the *Incentive Group* as local businesses sponsored the rewards, keeping the additional costs low. However, the 95% CIs surrounding this ICER are wide alluding to uncertainty in its value but from our CEAC at the £30,000/QALY cost-effectiveness threshold, it had an 85% chance of being cost-effective.

By calculating the population EVPI we were then able to quantify the uncertainty in this ICER. At the £30,000/QALY cost-effectiveness threshold the population EVPI was greater than £1.5 billion indicating that conducting further research would be cost-effective to decrease the uncertainty in our parameters, particularly our 'effect’ estimates. Indeed, a larger scale trial is currently being planned. However, our EVPI should be interpreted with caution since the standard approach to calculating EVPI requires a 'soft’ budget constraint and the assumption that health can continue to be purchased at a constant rate which can lead to an overestimated EVPI value [32]. Therefore our EVPI may not necessarily represent the maximum amount a healthcare system would be willing to pay for further research but it is so much larger than the anticipated costs of further research that the case for further research still holds.

### Employer’s perspective

From an employer’s perspective, the ICER was £2,700 per percentage increase in overall employee productivity which for different employers will have a different value depending on the size and success of their business. Essentially, if a business has revenue of at least £270,000 then the intervention will likely be cost-effective for them as they should get a return of at least equivalent to what they invested.

These findings broadly accord with the assumptions of the SLOTH time-budget model which categorizes all 24 hours of the day into five domains: **S**leep, **L**eisure, **O**ccupation, **T**ravel and **H**ome [33]. An employee’s decision about whether to exercise at work will depend upon the opportunity cost of their time taken out of their working day to exercise. This in turn depends upon what task they are displacing in order to exercise. If an employee has time during their lunch break to exercise then the opportunity cost may be lower than if they have to give up time spent working. Within the PAL study, we found the majority of participants chose to exercise between 12 noon and 2 pm i.e. during their lunch break. When employees are offered a financial incentive to do physical activity, as in the PAL scheme, their opportunity cost of doing exercise at work is decreased i.e. the incentive decreases the value employees place on their discretionary time at lunch, and they are effectively encouraged to perform exercise.

### Sustainable business model

A unique aspect of the loyalty card scheme, a financially incentivized physical activity program where rewards are sponsored from local businesses, is that it is based on a sustainable “business model” with a potential 'win-win’ situation for employers, local businesses and employees. In addition, this intervention should become more cost-effective as it is delivered to more participants as the intervention costs would increase minimally whilst the benefits would be much larger. These types of business partnerships used to promote physical activity support recent UK and US current government health initiatives, including the UK Public Health Responsibility Deal and the recently passed Patient Protection and Affordable Care Act in the US [4,5].

If the business sector is to be involved in tackling public health challenges, then the issue arises as to who should pay for these interventions. In the workplace many employers are now accepting responsibility for the health and wellbeing of their staff as they realize the potential economic benefits of a healthier and more active workforce [34]. Researchers have demonstrated that employees who have higher risk profiles are less productive at work, have higher rates of absenteeism and increased health care claims [35-40]. Therefore it has been suggested that since employers stand to benefit most economically from a healthier workforce, they should be prepared to pay for workplace health promotion programs.

### Future research and applications

Increases in physical activity have been associated with concurrent increases in health-related QOL [41,42]. For both groups we found small gains in utility, which using a previously validated algorithm could equate to small gains in productivity, with increasing physical activity levels. However, at baseline both groups started with high utility values of approximately 0.9. One of the issues associated with the EQ-5D utility index is that it cannot detect differences between health statuses at the high end of the utility range [43]. Therefore, the small and non-significant gains in utility (and productivity) we found would be in keeping with our relatively healthy study population at baseline. For future economic evaluations a new “capability” approach, is currently being developed which should be more sensitive at measuring the effects of public health interventions [44].

There is obviously a need for further research in this area as few studies investigating the cost-effectiveness of financial incentive programs exist. From CEAs of other workplace health promotion programs some valuable insights for improving the quality of future studies can be learned as many have been fraught with methodological limitations and accused of bias [45]. A recent systematic review on the financial return of workplace health promotion programs found a lack of randomized study design, few explicitly stated the perspective of their analysis or properly measured and valued costs and benefits, not all studies performed an incremental analysis of costs and benefits, and few studies conducted sensitivity analyses or reported on the uncertainty surrounding their cost-effectiveness estimates [45]. In addition, currently no 'gold standard’ guidelines exist to suggest what work related outcomes to measure and how, to incorporate them in a CEA. Therefore whilst our study addresses many of the above methodological limitations, it’s unclear if we should have measured productivity differently using a previously validated questionnaire such as the Productivity and Disease Questionnaire (PRODISQ) [46] or the World Health Organization Health and Work Performance Questionnaire (HPQ) [47] and possibly other work related outcomes such as employee turnover, improved employee satisfaction, and reduced accidents and injuries.

### Limitations of the study

When interpreting our CEA results it is important to highlight two main limitations. Firstly, a “do nothing” control group was not included in the PAL study and so we were unable to ascertain if just offering the opportunity to self monitor, as in the *No Incentive Group,* would have been cost effective on its own. However, evidence is available that 'self-monitoring’ is an effective technique for increasing physical activity levels [48,49] and other studies of internet enabled health promotion have been shown to be cost effective [27,50-53]. This might imply that our estimate of cost effectiveness for the *Incentive Group* is an underestimate of what it might have been if compared to a completely “do nothing” option. Secondly, we did not collect healthcare utilization data during our trial but since there was no significant difference found in absenteeism rates between our two groups, it is unlikely that healthcare costs would have been significantly different, especially over the short time frame of the study.

## Conclusion

By applying the traditional techniques of CEA we have demonstrated the potential for the PAL scheme to be cost-effective at improving adult physical activity levels and inducing greater gains in employee productivity and hence shown its economic value for both the health and employment sectors. However, further research is warranted to reduce the uncertainty surrounding our results. Of note, the PAL scheme is based on a sustainable “business model” which should become more cost-effective as it is delivered to more participants and can be adapted to suit other health behaviors and settings. This comes at a time when both UK and US governments are encouraging business involvement in tackling public health challenges.

## Abbreviations

QOL: Quality of life; QALY: Quality-adjusted life year; ICER: Incremental cost-effectiveness ratio; CEAC: Cost-effectiveness acceptability curve; EVPI: Expected value of perfect information; PAL: Physical activity loyalty card scheme; CEA: Cost-effectiveness analysis; RFID: Radio frequency identification; NFC: Near-field communication; GPAQ: General physical activity questionnaire; SPSS: Statistical package for social sciences; WTP: Willingness-to-pay; NICE: National institute for health and care excellence; PRODISQ: Productivity and disease questionnaire; HPQ: Health and work performance questionnaire.

## Competing interests

None of the authors have any competing interests.

## Authors’ contributions

MD: Assimilated all necessary data from the PAL intervention, performed the cost-effectiveness analysis, interpreted the results and drafted the manuscript. RH: Conceived of the study, supplied all data required from the PAL intervention and helped to draft the manuscript. MT: Was involved in the study design and reviewed the manuscript multiple times. KC: Conducted the probabilistic sensitivity analyses, value of information analyses and gave advice on all other statistical analyses performed. FK: Instructed on the study methodology and reviewed the manuscript multiple times. All authors read and approved the final manuscript.

## Pre-publication history

The pre-publication history for this paper can be accessed here:

http://www.biomedcentral.com/1471-2458/13/953/prepub

## Supplementary Material

Additional file 1: Table S1 A description of the financial incentives offered, their points’ and corresponding monetary value. Click here for file

Additional file 2: Table S2 Itemized costs of the PAL Scheme. Click here for file

## References

[B1] Kohl HW 3rd Craig CL Lambert EV Inoue S Alkandari JR Leetongin G Kahlmeier S The pandemic of physical inactivity: global action for public health Lancet 2012 380 294 305 10.1016/S0140-6736(12)60898-8 22818941

[B2] Scarborough P Bhatnagar P Wickramasinghe KK Allender S Foster C Rayner M The economic burden of ill health due to diet, physical inactivity, smoking, alcohol and obesity in the UK: an update to 2006–07 NHS costs J Public Heal 2011 33 527 535 10.1093/pubmed/fdr033 21562029

[B3] Chenoweth D Leutzinger J The economic cost of physical inactivity and excess weight in American adults J Phys Act Heal 2003 3 148 163 10.1123/jpah.3.2.148 28834464

[B4] Department of Health Healthy lives, healthy people. Our strategy for public health in England http://www.dh.gov.uk/en/Publicationsandstatistics/Publications/PublicationsPolicyAndGuidance/DH_121941

[B5] Kaiser Family Foundation Summary of new health reform lawhttp://www.kff.org/healthreform/8061.cfm

[B6] Silverman K DeFulio A Sigurdsson SO Maintenance of reinforcement to address the chronic nature of drug addiction Prev Med 2012 55 Suppl S46 S53 22668883 10.1016/j.ypmed.2012.03.013 PMC3437006

[B7] Sigmon SC Patrick ME The use of financial incentives in promoting smoking cessation Prev Med 2012 55 Suppl S24 S32 22525802 10.1016/j.ypmed.2012.04.007 PMC3411852

[B8] Higgins ST Washio Y Heil SH Solomon LJ Gaalema DE Higgins TM Bernstein IM Financial incentives for smoking cessation among pregnant and newly postpartum women Prev Med 2012 55 Suppl S33 S40 22227223 10.1016/j.ypmed.2011.12.016 PMC3399924

[B9] Finkelstein EA Brown DSDRDSDR Buchner DM A randomized study of financial incentives to increase physical activity among sedentary older adults Prev Med 2008 47 182 187 10.1016/j.ypmed.2008.05.002 18571226

[B10] Brown DS Finkelstein EA Brown DR Buchner DM Johnson FR Estimating older adults’ preferences for walking programs via conjoint analysis Am J Prev Med 2009 36 201 207 e4 10.1016/j.amepre.2008.10.014 19215845

[B11] Pope L Harvey-Berino J Burn and earn: A randomized controlled trial incentivizing exercise during fall semester for college first-year students Prev Med 2013 56 197 201 10.1016/j.ypmed.2012.12.020 23295170

[B12] Hunter RF Tully MA Davis M Stevenson M Kee F Physical activity loyalty cards for behavior change: a quasi-experimental study Am J Prev Med 2013 45 56 63 10.1016/j.amepre.2013.02.022 23790989

[B13] Higgins ST Silverman K Sigmon SC Naito NA Incentives and health: an introduction Prev Med 2012 55 Suppl S2 S6 22554884 10.1016/j.ypmed.2012.04.008 PMC4107351

[B14] Volpp KG Troxel AB Pauly MV Glick HA Puig A Asch DA Galvin R Zhu J Wan F DeGuzman J Corbett E Weiner J Audrain-McGovern J A randomized, controlled trial of financial incentives for smoking cessation N Engl J Med 2009 360 699 709 10.1056/NEJMsa0806819 19213683

[B15] Loyalty program http://en.wikipedia.org/wiki/Loyalty_program

[B16] Tully MA Hunter RF McAneney H Cupples ME Donnelly M Ellis G Hutchinson G Prior L Stevenson M Kee F Physical activity and the rejuvenation of Connswater (PARC study): protocol for a natural experiment investigating the impact of urban regeneration on public health BMC Public Health 2013 13 774 10.1186/1471-2458-13-774 24103381 PMC3844319

[B17] Hunter R Davis M Tully M Kee F Kostkova P, Szomszor M, Fowler D The Physical Activity Loyalty Card Scheme: Development and Application of a Novel System for Incentivizing Behaviour Change Electron Heal Lect notes Inst Comput Sci Soc Informatics Telecommun Eng 2012 170 177 Springer Berlin Heidelberg

[B18] Bull FC Maslin TS Armstrong T Global physical activity questionnaire (GPAQ): nine country reliability and validity study J Phys Act Heal 2009 6 790 804 10.1123/jpah.6.6.790 20101923

[B19] The EuroQol Group EuroQol–a new facility for the measurement of health-related quality of life Health Policy 1990 16 199 208 10109801 10.1016/0168-8510(90)90421-9

[B20] Ferrie JE Kivimaki M Head J Shipley MJ Vahtera J Marmot MG A comparison of self-reported sickness absence with absences recorded in employers’ registers: evidence from the Whitehall II study Occup Environ Med 2005 62 74 79 10.1136/oem.2004.013896 15657187 PMC1740949

[B21] Krol M Stolk E Brouwer W Predicting productivity based on EQ-5D: an explorative study Eur J Heal Econ 2013 [Epub ahead of print] 10.1007/s10198-013-0487-y 23761020

[B22] Brouwer WB Meerding WJ Lamers LM Severens JL The relationship between productivity and health-related QOL: an exploration Pharmacoeconomics 2005 23 209 218 10.2165/00019053-200523030-00002 15836003

[B23] Lamers LM Meerding WJ Severens JL Brouwer WB The relationship between productivity and health-related quality of life: an empirical exploration in persons with low back pain Qual Life Res 2005 14 805 813 10.1007/s11136-004-0800-4 16022073

[B24] NICE Guide to the Methods of Technology Appraisal 2008 London: NICE

[B25] Dolan P Gudex C Kind P Williams A A Social Tariff for EuroQol: Results from a UK General Population Survey, Discussion Paper No.138 1995 York: Centre for Health Economics, University of York

[B26] Briggs AH Wonderling DE Mooney CZ Pulling cost-effectiveness analysis up by its bootstraps: a non-parametric approach to confidence interval estimation Health Econ 1997 6 327 340 10.1002/(SICI)1099-1050(199707)6:4<327::AID-HEC282>3.0.CO;2-W 9285227

[B27] Van Wier MF Dekkers JC Bosmans JE Heymans MW Hendriksen IJ Pronk NP van Mechelen W van Tulder MW Economic evaluation of a weight control program with e-mail and telephone counseling among overweight employees: a randomized controlled trial Int J Behav Nutr Phys Act 2012 9 112 10.1186/1479-5868-9-112 22967224 PMC3499374

[B28] McCabe C Claxton K Culyer AJ The NICE cost-effectiveness threshold: what it is and what that means Pharmacoeconomics 2008 26 733 744 10.2165/00019053-200826090-00004 18767894

[B29] Fenwick E Claxton K Sculpher M The value of implementation and the value of information: combined and uneven development Med Decis Mak 2008 28 21 32 10.1177/0272989X07308751 18263559

[B30] Koerkamp BG Spronk S Stijnen T Hunink MG Value of information analyses of economic randomized controlled trials: the treatment of intermittent claudication Value Heal 2010 13 242 250 10.1111/j.1524-4733.2009.00656.x 19818058

[B31] Department of Enterprise Trade and Investment. Labour Market Statistics - June 2013 http://www.detini.gov.uk/statistical_press_release_-_june_2013__final_.pdf

[B32] McKenna C Chalabi Z Epstein D Claxton K Budgetary policies and available actions: a generalisation of decision rules for allocation and research decisions J Health Econ 2010 29 170 181 10.1016/j.jhealeco.2009.11.005 20018396

[B33] Pratt M Macera CA Sallis JF O’Donnell M Frank LD Economic interventions to promote physical activity: application of the SLOTH model Am J Prev Med 2004 27 3 Suppl 136 145 15450624 10.1016/j.amepre.2004.06.015

[B34] NICE Workplace Health Promotion: How to Encourage Employees to Be Physically Active 2008 London, UK: NICE

[B35] Burton WN Conti DJ Chen CY Schultz AB Edington DW The role of health risk factors and disease on worker productivity J Occup Environ Med 1999 41 863 877 10.1097/00043764-199910000-00007 10529942

[B36] Aldana SG Pronk NP Health promotion programs, modifiable health risks, and employee absenteeism J Occup Environ Med 2001 43 36 46 10.1097/00043764-200101000-00009 11201768

[B37] Wright DW Beard MJ Edington DW Association of health risks with the cost of time away from work J Occup Environ Med 2002 44 1126 1134 10.1097/00043764-200212000-00006 12500454

[B38] Goetzel RZ Long SR Ozminkowski RJ Hawkins K Wang S Lynch W Health, absence, disability, and presenteeism cost estimates of certain physical and mental health conditions affecting U.S. employers J Occup Environ Med 2004 46 398 412 10.1097/01.jom.0000121151.40413.bd 15076658

[B39] Schultz AB Edington DW Employee health and presenteeism: a systematic review J Occup Rehabil 2007 17 547 579 10.1007/s10926-007-9096-x 17653835

[B40] Mitchell RJ Bates P Measuring health-related productivity loss Popul Heal Manag 2011 14 93 98 10.1089/pop.2010.0014 PMC3128441 21091370

[B41] Bize R Johnson JA Plotnikoff RC Physical activity level and health-related quality of life in the general adult population: a systematic review Prev Med 2007 45 401 415 10.1016/j.ypmed.2007.07.017 17707498

[B42] Anokye NK Trueman P Green C Pavey TG Taylor RS Physical activity and health related quality of life BMC Public Health 2012 12 624 10.1186/1471-2458-12-624 22871153 PMC3490805

[B43] Kopec JA Willison KD A comparative review of four preference-weighted measures of health-related quality of life J Clin Epidemiol 2003 56 317 325 10.1016/S0895-4356(02)00609-1 12767408

[B44] Lorgelly PK Lawson KD Fenwick EA Briggs AH Outcome measurement in economic evaluations of public health interventions: a role for the capability approach? Int J Env Res Public Heal 2010 7 2274 2289 10.3390/ijerph7052274 PMC2898049 20623024

[B45] Van Dongen JM Proper KI van Wier MF van der Beek AJ Bongers PM van Mechelen W van Tulder MW Systematic review on the financial return of worksite health promotion programmes aimed at improving nutrition and/or increasing physical activity Obes Rev 2011 12 1031 1049 10.1111/j.1467-789X.2011.00925.x 21883870

[B46] Twisk JWR Applied Longitudinal Data Analysis for Epidemiology 2003 New York: Cambridge University Press

[B47] Murta SG Sanderson K Oldenburg B Process evaluation in occupational stress management programs: a systematic review Am J Heal Promot 2007 21 248 254 10.4278/0890-1171-21.4.248 17375490

[B48] Michie S Abraham C Whittington C McAteer J Gupta S Effective techniques in healthy eating and physical activity interventions: a meta-regression Heal Psychol 2009 28 690 701 10.1037/a0016136 19916637

[B49] Williams DM Matthews CE Rutt C Napolitano MA Marcus BH Williams DM Interventions to increase walking behavior Med Sci Sports Exerc 2008 40 7 Suppl) s567 s573 18562974 10.1249/MSS.0b013e31817c7006 PMC2694671

[B50] Rasu RS Hunter CM Peterson AL Maruska HM Foreyt JP Economic evaluation of an Internet-based weight management program Am J Manag Care 2010 16 e98 e104 20370312

[B51] Misra S Lairson DR Chan W Chang YC Bartholomew LK Greisinger A McQueen A Vernon SW Cost effectiveness of interventions to promote screening for colorectal cancer: a randomized trial J Prev Med Public Heal 2011 44 101 110 10.3961/jpmph.2011.44.3.101 PMC3249245 21617335

[B52] Krukowski RA Tilford JM Harvey-Berino J West DS Comparing behavioral weight loss modalities: incremental cost-effectiveness of an internet-based versus an in-person condition Obes (Silver Spring) 2011 19 1629 1635 10.1038/oby.2010.341 PMC3137759 21253001

[B53] Maloney S Haas R Keating JL Molloy E Jolly B Sims J Morgan P Haines T Breakeven, cost benefit, cost effectiveness, and willingness to pay for web-based versus face-to-face education delivery for health professionals J Med Internet Res 2012 14 e47 10.2196/jmir.2040 22469659 PMC3376523

