# Supplementary material for: A lesson in business: cost-effectiveness analysis of a novel financial incentive intervention for increasing physical activity in the workplace
Source: BMC Public Health. 2013 Oct 10;13:953. doi: 10.1186/1471-2458-13-953 (PMC3852549; doi:10.1186/1471-2458-13-953)
Supplement: Additional file 1: Table S1 — A description of the financial incentives offered, their points’ and corresponding monetary value. [file 1471-2458-13-953-S1.docx]

Additional file 1: Table S1

A description of the financial incentives offered, their points’ and corresponding monetary value [12].

| **Description of Incentive** | **PAL Points Value** | **Monetary Value** |
| --- | --- | --- |
| Sandwich (Buy 1 get 1 free) | 75 pts | £2.50 |
| Exercise class pass | 120 pts | £4.00 |
| Cinema pass | 150 pts | £5.00 |
| Session of bowling / skating or indoor playground | 150 pts | £5.00 |
| £5 Spa & Beauty voucher off any treatment | 150 pts | £5.00 |
| £10 Sports shop voucher | 300 pts | £10.00 |
| £10 5-a-side pitch hire voucher | 300 pts | £10.00 |
| Cinema pass x 2 | 300 pts | £10.00 |
| £10 Spa & Beauty voucher off any treatment | 300 pts | £10.00 |
| Exercise class pass x 5 | 600 pts | £20.00 |
| £20 5-a-side pitch hire voucher | 600 pts | £20.00 |
| Cinema pass x 4 | 600 pts | £20.00 |
| £20 Spa & Beauty voucher off any treatment | 600 pts | £20.00 |
| Personal training session | 750 pts | £25.00 |
| Exercise class pass x 10 | 960 pts | £32.00 |
| 5-a-side pitch hire for 1 hour | 1200 pts | £40.00 |
| One month gym membership for you & a guest | 1800 pts | £60.00 |
| **Total** |  |  |
