# Supplementary material for: A lesson in business: cost-effectiveness analysis of a novel financial incentive intervention for increasing physical activity in the workplace
Source: BMC Public Health. 2013 Oct 10;13:953. doi: 10.1186/1471-2458-13-953 (PMC3852549; doi:10.1186/1471-2458-13-953)
Supplement: Additional file 2: Table S2 — Itemized costs of the PAL Scheme. [file 1471-2458-13-953-S2.docx]

Additional file 2: Table S2

Itemized costs of the PAL Scheme.

| **Resource** | **Description** | **Unit Cost** | **Quantity** | **Total Cost** |
| --- | --- | --- | --- | --- |
| **Website Development** | \| IT Consultant-built and maintained website (www.palcard.co.uk). \| \| --- \| \|  \| | £13.33/hr | 236.5hrs | £3,153 |
|  | Research Fellow - website design and day-to-day management of loyalty card scheme. | AC2/sp33 | 3mths/  0.25 FTE | £8225 |
| **Software** | \| CredX software and database to monitor all physical activity. \| \| --- \| \| Web service support provided for building and hosting web interface.  (CredX is designed and manufactured by Intelligent Health, a company which designs IT systems to monitor physical activity. [www.intelligenthealth.co.uk](http://www.intelligenthealth.co.uk).) \| |  |  | £3,150 |
|  | CredX license fee. |  |  | £1,000 |
| **Hardware** | 5 outdoor CredX sensors and 2 indoor sensors. | £900 | 7 | £6,300 |
|  | 500 branded PAL cards. | £1.50 | 500 | £750 |
|  | Consultancy & travel. |  |  | £4,200 |
|  | Installation of sensors by grounds men. | £39.00 + VAT @ 20% |  | £46.80 |
| **Intervention running costs** | Maintenance of sensors- performed as part of regular maintenance of the workplace grounds by Belfast City Council. | - | - | - |
|  | Delivery of PAL cards to participants. | 40p/mile | 13 miles x6 | £31.20 |
| **Total** |  |  |  | £26,856 |

Additional costs for the Incentive group only.

| **Resource** | **Description** | **Unit Cost** | **Quantity** | **Total Cost** |
| --- | --- | --- | --- | --- |
| **Vouchers** | Marketing consultant hired to negotiate ‘in-kind’ vouchers from local businesses | £45/hr+  VAT@20% | 75.5 hrs | £4,077 |
| **Intervention running costs** | Delivery of vouchers to participants & administration time | 40p/mile | 13 miles  X4 | £20.80 |
| **Total** |  |  |  | £4,098 |
